# Vulvar Hidradenoma Papilliferum Dermoscopically Mimicking Basal Cell Carcinoma

**DOI:** 10.5826/dpc.1104a70

**Published:** 2021-10-01

**Authors:** Andrija Jovic, Danijela Popovic, Sladjana Cekic, Natasa Vidovic, Nikola Zivkovic, Zorana Zlatanovic, Danica Tiodorovic

**Affiliations:** 1 Clinic of Dermatovenereology, Clinical Center of Nis, Serbia; 2 Center for Pathology and Pathological Anatomy, Clinical Center of Nis, Serbia; 3 Faculty of Medicine, University of Nis, Nis, Serbia

**Keywords:** hidradenoma papilliferum, dermoscopy, vulvar basal cell carcinoma, differential diagnosis, adnexal tumor

## Introduction

Hidradenoma papilliferum (HP), otherwise known as papillary hidradenoma, is a benign adnexal neoplasm that almost exclusively occurs in the adult female affecting the anogenital area. HP can infrequently appear in ectopic extragenital sites including the head, chest, and abdomen, affecting both females and males equally. Although it represents the most common vulvar adnexal neoplasm, dermoscopic features of vulvar HP have been sparsely reported, being limited to only one small case series [1]. Herein, we present a case of HP with peculiar dermoscopic features resembling non-pigmented basal cell carcinoma.

## Case Presentation

**Figure 1:**
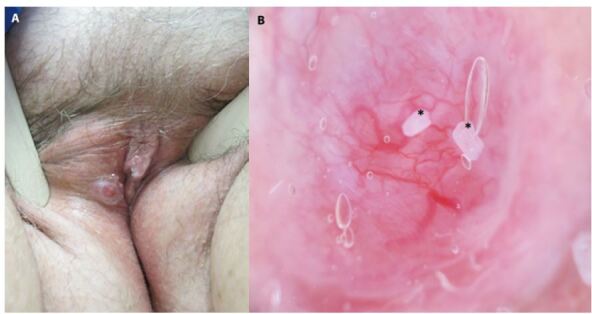
Clinical and dermoscopic aspects of vulvar hidradenoma papilliferum (HP). (A) HP involving the right inner site of the labia majora in a form of a red nodule with non-ulcerated surface. (B) Dermoscopy revealed prominent and well-focused arborizing vessels over the pinkish and whitish background. (*) Artifacts.

**Figure 2:**
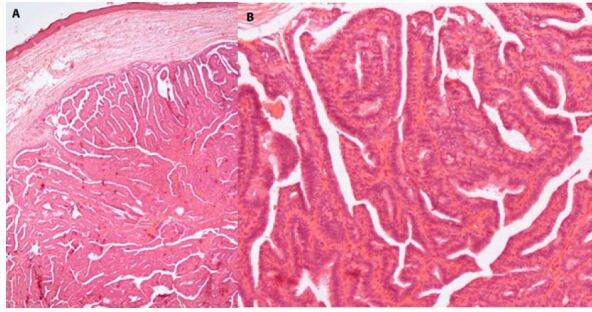
(A) Histopathological examination revealed a well-circumscribed dermal tumor without connection with the overlying non-ulcerated epidermis (H&E, ×4). (B) Tubular and papillary structures lined by epithelium consisted of the layers of two cell types: cuboidal and luminal columnar cells (H&E, ×10).

A 68-year-old female patient presented with a 9-month history of an asymptomatic, slow-growing nodule on the right inner side of the labia majora. Her past medical history was unremarkable. Clinical examination revealed a firm, reddish, non-ulcerated nodular lesion measuring 25×10 mm in diameter (Figure 1A). Upon dermoscopy, the lesion showed a prominent vascular pattern consisting of the well-focused arborizing vessels over the pinkish background with whitish areas (Figure 1B). The lesion was completely excised, and histopathological examination was consistent with the diagnosis of hidradenoma papilliferum revealing a characteristic, well-circumscribed dermal lesion with prominent elongated tubular and papillary structures lined by columnar cells without cellular atypia and mitoses (Figure 2, A and B). The patient was reassured about the benign nature of the lesion.

## Conclusion

Vulvar HP are rare benign adnexal lesions that may appear with different clinical presentations, with the most common one being unilobular, a small nodule ranging from 10 mm to 20 mm, while other clinical forms including the giant one, multilobular or plaque type are considered as rare. With respect to color, vulvar HP are predominantly red or skin-colored, usually revealing a smooth and non-ulcerated surface. Furthermore, the lesions with blue coloration have also been reported. A differential diagnosis based on a clinical presentation is broad and often involves both benign and malignant lesions, including epidermal cyst, blue nevus, squamous cell carcinoma, melanoma, or basal cell carcinoma, particularly in the presence of ulceration [1].

Currently, there is a lack of published data regarding the dermoscopic features seen in vulvar HP and to the best of our knowledge, only one case series study reported dermoscopy findings of this rare neoplasm [1]. Tosti et al [1] presented a series of histopathologically proven vulvar HPs, describing the dermoscopic features in 5 out of 7 cases. According to them, vulvar HPs are typified by a dermoscopic variability, whereas the presence of the polymorphous vascular pattern was the most prevalent dermoscopic feature. Namely, 4 out of 5 lesions exhibited a polymorphous vascular pattern comprising lacunae (red globular structures), glomerular, linear, serpentine and telangiectatic vessels. However, in this case, we observed a monomorphous vascular pattern composed of well-focused arborizing vessels similar to those found in the vulvar non-pigmented basal cell carcinoma (BCC) described by Cinotti et al [2]. Furthermore, we observed homogenous whitish areas in which there is another dermoscopically overlapping finding of genital BCC [2].

In conclusion, we report a peculiar case of vulvar HP revealing overlapping dermoscopic features with non-pigmented BCC features. Additional studies, including a larger number of vulvar HPs, may better characterize the dermoscopic features and provide potential dermoscopic clues for this rare benign lesion.
